# Left ventricular hypertrophy, carotid atherosclerosis, and cognitive impairment in peritoneal dialysis patients

**DOI:** 10.1186/s12872-023-03130-0

**Published:** 2023-03-09

**Authors:** Xuejing Zhu, Ran Jing, XiaoPing Li, Wanfen Zhang, Yushang Tang, Tongqiang Liu

**Affiliations:** Department of Nephrology, The Affiliated Changzhou No.2 People’s Hospital of Nanjing Medical University, 68# Middle Gehu Road, Changzhou, 213164 Jiangsu Province People’s Republic of China

**Keywords:** Peritoneal dialysis, Left ventricular hypertrophy, Carotid atherosclerosis, Cognitive impairment

## Abstract

**Background:**

Left ventricular hypertrophy (LVH) and carotid atherosclerosis (CAS) have been identified as factors associated with cognitive impairment (CI) but have not been studied in patients undergoing peritoneal dialysis (PD). This study investigated the relationship between LVH and CAS and cognitive function in patients undergoing PD.

**Methods:**

In this single-center cross-sectional study, the clinically stable patients who were over 18 years of age and had undergone PD for at least 3 months were enrolled. Cognitive function was assessed using the Montreal Cognitive Assessment (MoCA), which included seven areas: visuospatial/executive function, naming, attention, language, abstraction, delayed recall, and orientation. LVH was defined as LVMI > 46.7 g/m^2.7^ in women and LVMI > 49.2 g/m^2.7^ in men. CAS was defined as carotid intima-media thickness ≥ 1.0 mm and/or the presence of plaque.

**Results:**

A total of 207 patients undergoing PD were recruited, with an average age of 52.14 ± 14.93 years and a median PD duration of 8 months (5–19 months). The CI rate was 56%, and the prevalence of CAS was 53.6%. LVH occurred in 110 patients (53.1%). Patients in the LVH group tended to be older, and had a higher body mass index, a higher pulse pressure, a higher male proportion, a lower ejection fraction, a higher prevalence of cardiovascular disease and CI, and a lower MoCA scores.Multivariate logistic regression analysis was conducted to analyze the association between LVH and CI (OR, 10.087; 95% confidence interval, 2.966–34.307). And the association between LVH and CI was still supported after propensity matching scores. CAS was not significantly associated with CI.

**Conclusion:**

LVH is independently associated with CI in patients undergoing PD, while CAS is not significantly associated with CI.

**Supplementary Information:**

The online version contains supplementary material available at 10.1186/s12872-023-03130-0.

## Introduction

Peritoneal dialysis (PD), also known as home PD, requires patients to be able to self-supervise and self-manage [1], which to a certain extent depends on normal cognitive function [2]. Unfortunately, according to relevant studies, the incidence of cognitive impairment (CI) in this population is 28–66% [3]. CI increases the incidence of complications such as peritonitis, cardiovascular and cerebrovascular disorders, and calcium and phosphorus metabolism disorders in dialysis patients and increases the hospitalization rate and mortality of patients [4–6]. CI is also recognized as an independent predictor of survival in dialysis patients. Therefore, there is an urgent need to explore and identify the risk factors leading to CI in dialysis patients and perform active intervention.

There are many risk factors for CI in patients undergoing PD, including general demographic characteristics (age, gender, socioeconomic status, and education level), traditional factors (such as hypertension, diabetes [7, 8], and cardiovascular risk factors [9–12]), non-traditional factors (such as hyperparathyroidism, anemia, inflammation [13], and malnutrition [14]), and uremia- or dialysis-related factors [15, 16]^.^ Among them, cardiovascular risk factors are also the main cause of death in patients undergoing PD [17–19]. Left ventricular hypertrophy (LVH) and carotid atherosclerosis (CAS) are major predictors of cardiovascular disease (CVD) [20]^.^ In recent years, Elias and Kaffashian et al. showed that LVH was negatively correlated with cognitive function [21–25], and the association might not only be indirect. LVH independently leads to cognitive decline and CI [26–32], and hemodialysis patients with CVDs have worse processing speed and executive function [9]. One possible mechanism underlying the relationship between LVH and CI is the presence of white matter damage [33]. However, there is still a lack of research on the correlation between LVH and cognitive function in patients undergoing PD.

In addition, CAS includes carotid stiffness, increased carotid intima-media thickness, carotid stenosis, and carotid plaque characteristics [34, 35]. Although traditionally thought to primarily cause ischemic stroke, there is an increasing body of evidence showing that carotid atherosclerosis contributes to the development of CI and dementia. It has been shown that atherosclerosis is an independent risk factor for CI [35–45]_._ However, there is still a lack of relevant studies on patients undergoing PD.

Therefore, this cross-sectional study aimed to investigate whether LVH and CAS were independently associated with CI during PD.

## Materials and methods

### Study design and participants

This study was a cross-sectional observational survey of patients undergoing PD. The study has been approved by the Ethics Committee of Changzhou Second People's Hospital. From January 2017 to July 2022, patients undergoing continuous ambulatory peritoneal dialysis (CAPD) hospitalized in the Department of Nephrology, Changzhou Second Hospital Affiliated to Nanjing Medical University were screened.

Inclusion criteria were as follows: age > 18 years; undergoing CAPD for ≥ 3 months and clinically stable; and able to undergo all measurements and fill in all questionnaires. Exclusion criteria were as follows: kidney transplantation, systemic infections, acute cardiovascular events, active hepatitis, or cancer; surgery or trauma in the month before the study; and any neurological conditions such as dementia or Parkinson's disease, a history of mental illness such as depression or schizophrenia, and any other serious health conditions such as malignancy, severe infections, or impairments in vision, hearing, speech, or comprehension skills.

All participants received a conventional glucose-based lactate-buffered PD solution.

### Clinical characteristics

Demographic characteristics and complications were recorded, including age, sex, body mass index (BMI), education level, dialysis duration, systolic blood pressure, diastolic blood pressure, pulse pressure, smoking, diabetes mellitus, hypertension, primary kidney disease, and history of CVD. Education level was recorded as the highest school level for which a diploma was obtained: primary school or below, middle school, high school and beyond. Primary kidney disease includes chronic glomerulonephritis, diabetic nephropathy, and others (such as IgA nephropathy, nephrotic syndrome, lupus nephritis, Sjogren's syndrome, and ANCA-associated vasculitis). CVD information was obtained from medical history reviews, and CVD was recorded if any of the following conditions were present: angina pectoris, grade III or IV congestive heart failure (as classified based on the guidelines of the New York Heart Association), transient ischemic attack, myocardial infarction, and cerebrovascular accident.

### Laboratory methods

Blood samples were collected after overnight fasting while continuing PD treatment, and relevant indicators were determined using standardized equipment and procedures, including serum albumin, AST, ALT, triglyceride, total cholesterol, low-density lipoprotein, high-density lipoprotein, phosphorus, calcium, sodium, potassium, parathyroid hormone, urea nitrogen, creatinine, uric acid, glycosylated hemoglobin, fasting blood glucose, high-sensitivity C-reactive protein (hsCRP), and hemoglobin. To rule out cognitive decline secondary to nutritional or endocrine changes, thyroid hormone levels in the blood were measured by standard laboratory methods. Dialysis adequacy was defined based on total Kt/V and creatinine clearance (Ccr).

### Echocardiographic measurements

Echocardiography was performed by trained sonographers at our hospital using a Philips iE33 Doppler echocardiography system with a real-time 3D probe X3-1 (frequency 1–3 MHz) (Philips, Best, The Netherlands). Left ventricular end-diastolic diameter (LVDd), septal thickness (IVST), left ventricular posterior wall thickness (LVPWT), and ejection fraction (EF) were measured. All the above data were averaged over three cardiac cycles.

Left ventricular mass (LVM) was estimated by the formula of Devereux et al.: LVM (g) = 0.8 × 1.04 [(LVID + IVS + PWT)^3^ − (LVIDd)^3^] + 0.6.LVM was normalized for body height to the 2.7 (LVMI). LVH was defined as LVMI > 46.7 g/m^2.7^ in women and LVMI > 49.2 g/m^2.7^ in men [46].

Cardiac parameter, Relative wall thickness (RWT), was calculated as the ratio of twice the posterior wall thickness divided by the left ventricular internal diameter in diastole, and a value over 0.42 cm was defined as an elevated RWT [47].Four categories of left ventricular geometry were defined: (1) normal (normal RWT and LVMI); (2) eccentric hypertrophy (normal RWT and high LVMI); (3) concentric hypertrophy (high RWT and high LVMI); and (4) concentric remodeling (high RWT and normal LVMI).

### Carotid ultrasound measurement

Echocardiography was performed by a well-trained sonographer in our hospital using a Sonos5500 color Doppler ultrasound diagnostic instrument with a probe frequency of 3–11 MHz. The patient was placed in the supine position, and transverse 2D images of the bilateral common carotid artery, the extracranial segment of the internal carotid artery, the external carotid artery, and the carotid bifurcation were acquired segment by segment, and the intima of the wall and the presence of plaque were observed. According to guidelines of the American Heart Association, CAS is defined as carotid intima-media thickness ≥ 1.0 mm and/or the presence of plaque [48–50].

### Cognitive function measurement

All participants were tested for cognitive function using the Chinese version of the Montreal Cognitive Assessment (MoCA), which assesses seven cognitive domains, namely visuospatial/executive functioning, naming, attention, language, abstraction, delayed recall, and orientation. For patients with less than 12 years of education, MoCA scores were increased by 1 point to correct for the bias caused by educational level. Possible scores range from 0 to 30, with higher scores indicating better cognitive status. CI was defined as a total MoCA score of < 26, and a score of ≥ 26 was considered to indicate normal cognition.

Assessments of cognitive function were performed in a separate room with 1 medical staff to 1 patient. In total, 4 medical staff members participated in this study as observers and all completed a training program that taught them the methods and processes to ensure the integrity and accuracy of the assessment.

### Statistical analyses

SPSS version 25.0 and GraphPad Prism 9.4.1 (681) were used for statistical analysis and plotting. Normally distributed continuous variables are presented as mean and standard deviation. Non-normally distributed continuous variables are presented as medians and interquartile ranges. Categorical variables are expressed as frequencies and percentages.

The independent sample *t*-test, the Mann–Whitney U test, and the chi-square test were used to compare the differences of continuous variables and categorical variables between the two groups. All variables with a significance level of *P* < 0.10 on the univariate test were included in further multivariate analyses. Three linear regression models were then developed. Model 1 was the basic model, including LVH and accepted demographic data (age, sex, BMI, and education level). Model 2 was adjusted for cardiovascular risk factors (diabetes mellitus, CVD, common CAS, smoking history, beta-blockers, pulse pressure, and EF) based on Model 1. Model 3 was adjusted for laboratory parameters (hemoglobin, albumin, hsCRP, and Ccr) based on Model 2. Next, the risk factors of CI were analyzed by multivariate logistic regression analysis.

To reduce the effect of possible selection bias, the effect of the small number of patients with LVH, and the effect of the relatively large number of associations on the reliability of the multivariable model and to adjust for the effects of other potential confounders without reducing the ratio of events per variable, we further performed sensitivity analyses for LVH by propensity score matching. Propensity score matching was performed using the following variables: age, sex, BMI, RRF, CVD, pulse pressure, and EF. The maximum difference in propensity scores that allowed matching was 0.02, and all test levels were two-sided, with *P* < 0.05 indicating statistical significance.

## Results

### Basic characteristics

Of 268 patients undergoing PD screened for inclusion in this study, 207 (77.2%) agreed to participate and completed the MoCA questionnaire. The general characteristics of our participants were as follows: age, 52.14 ± 14.93 years; BMI, 22.99 ± 3.66; and dialysis duration, 8 months (5–19 months). Males accounted for 51.2% of the study population. Of the study population, 96.1% had hypertension and 29% had diabetes. The prevalence of LV hypertrophy as assessed by echocardiography was 53.1%. The prevalence of CAS was 53.6%.

### Clinical features of left ventricular hypertrophy and carotid atherosclerosis

Subjects were grouped according to LVH. As shown in Table 1, the comparison of demographic and biochemical data revealed significant differences in age, sex, BMI, CVDs, pulse pressure, and EF between the two groups. Age, pulse pressure, and CVD were higher in the LVH group than in the non-LVH (NLVH) group. The EF was lower in the LVH group than in the NLVH group. Other variables including antihypertensive drugs, hypertension, diabetes, triglycerides, total cholesterol, and CAS did not differ significantly (Table 1).

**Table 1:** Demographic and cardiovascular data were compared between LVH and NLVH groups

Characteristics	NLVH (n = 97)	LVH (n = 110)	T/Z/X^2^	*P*
Age (years)	48.36 ± 14.82	55.46 ± 14.27	− 3.509	0.001
BMI (kg/m^2^)	22.41 ± 4.07	23.55 ± 3.14	− 2.261	0.025
Males n (%)	41 (42.3)	65 (31.4)	5.839	0.016
Diuretics n (%)	13 (13.4)	11 (10)	0.582	0.446
Beta-blockers n (%)	48 (49.5)	50 (45.5)	0.336	0.562
Calcium channel blockers n (%)	80 (82.5)	99 (90)	2.496	0.114
ACE/ARB inhibitors n (%)	18 (18.6)	16 (14.5)	0.604	0.437
Cardiovascular disease n (%)	6 (6.2)	19 (17.3)	5.967	0.015
Hypertension n (%)	92 (94.8)	107 (97.3)	0.817	0.366
Diabetes mellitus n (%)	23 (23.7)	36 (32.7)	2.056	0.152
Smoking History n (%)	38 (39.2)	55 (50)	2.441	0.118
Systolic BP (mmHg)	141.05 ± 23.07	146.9 ± 19.49	− 1.977	0.050
Diastolic BP (mmHg)	87.12 ± 15.05	86.49 ± 12.06	0.331	0.741
Pulse pressure (mmHg)	53.93 ± 15.6	60.41 ± 15.1	− 3.034	0.003
Total cholesterol, (mmol/L)	4.37 ± 1.01	4.38 ± 0.99	− 0.101	0.92
triglyceride, (mmol/L)	1.3 (0.93–1.88)	1.35 (0.87–1.83)	− 0.116	0.907
HDL cholesterol (mmol/L)	1 (0.88–1.26)	0.98 (0.80–1.2)	− 1.473	0.141
LDL cholesterol (mmol/L)	2.08 ± 0.68	1.99 ± 0.65	0.954	0.341
LV ejection fraction (%)	60 (56.5–63)	57 (53–61)	− 3.782	< 0.001
CAS n (%)	47 (48.5)	64 (58.2)	1.962	0.161

Values for categorical variables are given as number (percentage); values for continuous variables, as mean ± standard deviation or median [interquartile range]

*ACE* Angiotensin Converting Enzyme; *ARB* Angiotensin Receptor Blocker; *BMI* Body mass index; *BP*, Blood pressure; *CAS* Carotid atherosclerosis; *HDL* High-density lipoprotein; *LDL* Low-density lipoprotein; *LVMI* Left ventricular Mass Index; *LVH* Left ventricular hypertrophy; *NLVH* Non Left ventricular hypertrophy; *LV* Left ventricle

Participants were divided into two groups according to the occurrence of CAS. As shown in Additional file 1: Table S1, there were significant differences in the use of beta-blockers and the proportion of people smoking between the two groups, while there were no significant differences in other variables including age, BMI, gender, hypertension, diabetes, and antihypertensive drugs (Additional file 1: Table S1).

### Left ventricular hypertrophy, carotid atherosclerosis, and cognitive function

**Fig. 1:**
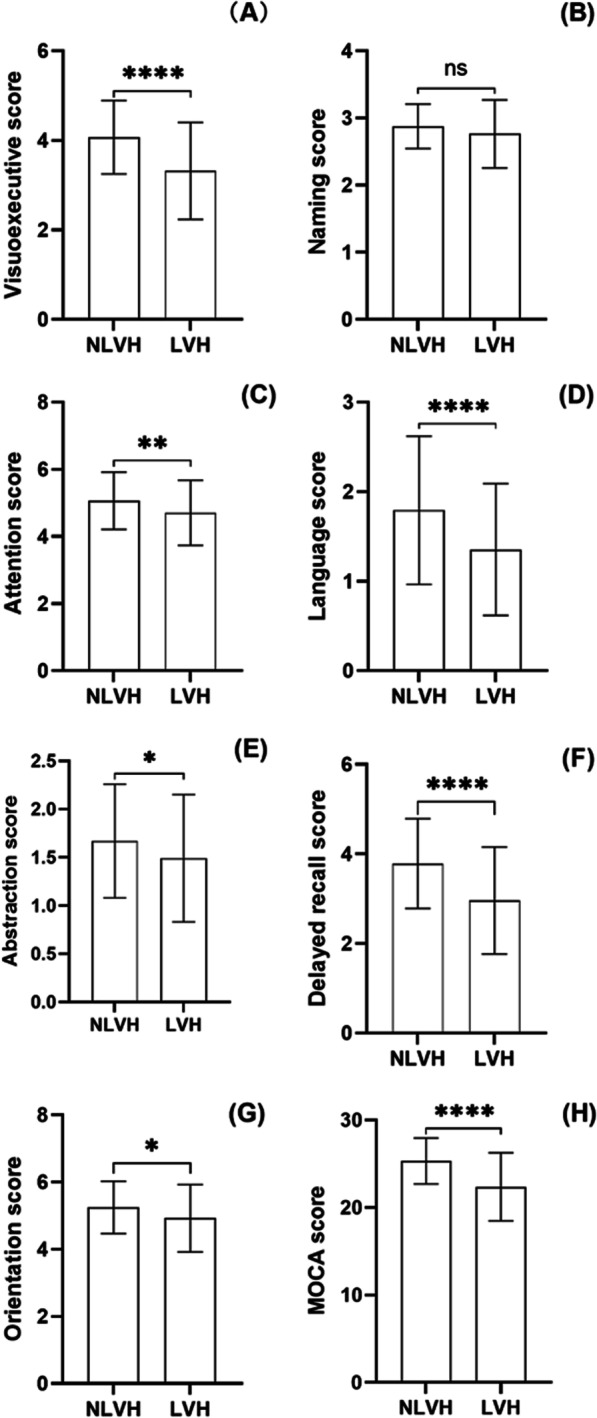
Comparison of cognitive domains between LVH and NLVH groups. *Note*: **** = *P* < 0.001.** = *P* < 0.01.* = *P* < 0.05. ns = no significance. Abbreviations: *NLVH* Non-left ventricular hypertrophy, *LVH* Left ventricular hypertrophy

In the present study, the prevalence of CI was 56%. The incidence of CI was significantly higher in the LVH group (74.5%) than in the NLVH group (35.1%) (*P* < 0.001). In addition, the total MoCA score in the LVH group was significantly lower than that in the NLVH group (Fig. 1H). In addition, there were significant differences in visuospatial/executive function (*P* < 0.001) (Fig. 1A), language (*P* < 0.001) (Fig. 1D), delayed recall (*P* < 0.001) (Fig. 1F), attention (*P* = 0.007) (Fig. 1C), abstract function (*P* = 0.028) (Fig. 1E), and orientation (*P* = 0.026) (Fig. 1G). There was no significant difference in naming (*P* = 0.119) (Fig. 1B).

In addition, in the left ventricular geometry, there were 79 cases (38.2%) in the normal group, 52 (25.1%) in eccentric hypertrophy, 58 (28.0%) in centripetal hypertrophy, and 19 (9.2%) in centripetal remodeling. The incidence of cognitive impairment in centripetal hypertrophy (79.3%) was slightly higher than that in centripetal hypertrophy (69.2), and the difference was not statistically significant (*P* > 0.05) (Table 2).

**Table 2:** Differences in left ventricular geometry and cognitive impairment in peritoneal dialysis patients

	Normal (N = 78)	Eccentric hypertrophy (N = 52)	Concentric hypertrophy (N = 58)	Concentric remodeling (N = 19)	*P*
CI,n (%)	24 (30.8)	36 (69.2)^ad^	46 (79.3)^be^	10 (52.6)^cf^	< 0.001

Values for categorical variables are given as number (percentage)

*C*I Cognition Impairment

^a^*P* < 0.001, Normal group versus Eccentric hypertrophy group

^b^*P* < 0.001, Normal group versus Concentric hypertrophy group

^c^*P* < 0.05, Normal group versus Concentric remodeling group

^d^*P* > 0.05, Eccentric hypertrophy group versus Concentric hypertrophy group

^e^*P* > 0.05,Eccentric hypertrophy group versus Concentric remodeling group

^f^*P* < 0.01, Concentric hypertrophy group versus Concentric remodeling group

In the CAS group, there was no significant difference in the total MoCA score between the two groups (*P* = 0.152), but there was a significant difference in the proportion of CI between the two groups (*P* = 0.014).

In the subgroup with normal cognitive function, patient demographics, laboratory data, and comorbidities (age, education, diabetes, hemoglobin, albumin, CRP, CAS percentage, Ccr, LVMI, and LVH percentage) were significantly different compared with the CI group (Additional file 2: Table S2). All these variables and the influencing factors of LVH and CAS were included in the subsequent analysis as recognized confounding factors. Three models were established by multivariate logistic regression analysis. Model 1 included LVH and demographic data (age, BMI, sex, and education); Model 2 was adjusted for cardiovascular risk factors (diabetes mellitus, CVD, smoking history, beta-blockers, pulse pressure, EF, and CAS) based on Model 1. Model 3 was adjusted for laboratory indicators (hemoglobin, albumin, and CRP) based on Model 2. It was found that the association between LVH and CI was not weakened (Table 3). Model 4 was established after propensity score matching (Table 4). After adjusting for age, education level, diabetes mellitus, hemoglobin, albumin, hsCRP, and Ccr, LVH was still independently associated with CI in Model 4.

**Table 3:** Association of left ventricular hypertrophy with cognitive impairment by univariable and multivariable logistic regression analysis

Variable	LVH	
	OR (95% CI)	*P* Value
Univariable	5.426 (2.983–9.871)	< 0.001
Multivariable model 1	6.148 (2.785–13.569)	< 0.001
Multivariable model 2	6.291 (2.700–14.657)	< 0.001
Multivariable model 3	10.087 (2.966–34.307)	< 0.001

Multivariable model 1: adjusted for demographic and clinical measures (including age, sex, body mass index, level of education). Model 2: model 1plus cardiovascular risk factors (including diabetes mellitus, cardiovascular disease, smoking history, beta-blockers, pulse pressure, CAS and LV ejection fraction) Model 3: model 2plus laboratory measures (including hemoglobin, albumin, Ccr and high-sensitivity C-reactive protein). *OR* Odds ratio; *95% CI* 95% Confidence interval

**Table 4:** Univariate and multivariate logistic regression analyses after propensity score matching showed an association between left ventricular hypertrophy and cognitive impairment

Variable	LVH	
	OR (95% CI)	*P* Value
Univariable	4.312 (2.109–8.816)	< 0.001
Multivariable model 4	9.214 (2.039–36.766)	0.002

Model 4: adjusted for age, level of education, diabetes mellitus, hemoglobin, albumin and high-sensitivity C-reactive protein after a propensity score match. The following variables were used for propensity score matching: age, sex, body mass index, cardiovascular disease, pulse pressure, LV ejection fraction. *OR* Odds ratio; *95% CI* 95% confidence interval

In addition, after adjusting for LVH and all confounding factors, a regression model was constructed (Additional file 3: Table S3), and LVH was independently associated with CI in patients undergoing PD (*P* < 0.001). CAS was not an independent risk factor for CI.

## Discussion

In the present study, in addition to established risk factors such as age and education level, LVH was found to be a new potential risk factor for CI in patients undergoing PD, especially with significant differences in visuospatial/executive function, delayed memory, language, and attention. CAS is not an independent risk factor for CI in patients undergoing PD.

The prevalence of CI based on MoCA scores in this PD population was 56%, which is similar to the percentages reported by Salazar-Felix [51] and Yi et al. [52] (65% and 49.9%, respectively). In addition, a systematic review and meta-analysis showed that the prevalence of CI in patients undergoing PD ranged from 3.3% to 74.5% [53], which could be attributed to the different neuropsychological tests used. Nasreddine et al. [54] found that compared with MMSE, MoCA had a higher sensitivity in detecting early CI (78% and 18%) and had good specificity (87%) and positive and negative predictive values (89% and 91%, respectively). Moreover, it has been reported that the MoCA test is more sensitive in assessing the cognitive ability of patients undergoing PD [54], especially in visuospatial/executive function and language.

In epidemiological studies, cardiovascular risk factors are the leading cause of death in patients undergoing PD, but an accurate prediction based on comprehensive risk factors remains challenging. LVH is a typical sign of cardiac end-organ damage, and LVM is the comprehensive temporal correlation between high blood pressure and other cardiovascular risk factors, which can be used as a marker of the chronicity and degree of elevated blood pressure, as well as an indicator of the long-term burden of vascular risk factors [21]. In addition, multiple meta-analyses [29, 30, 32, 55] have shown a positive correlation between LVH and CI. Therefore, we hypothesized that there would be such an association in patients undergoing PD. In the present study, the proportion of LVH in patients undergoing PD with CI was significantly higher than that in the non-CI (NCI) group, and LVH remained an independent predictor of CI after adjusting for demographic data, cardiovascular risk factors, laboratory parameters, and propensity score matching.

In end-stage renal disease (ESRD) treated by dialysis, fluid overload and arterial hypertension often contribute to a combination of eccentric and concentric hypertrophy, which may be influenced both by inadequate volume and blood pressure control [56]. In a study [57], 86.4% of the patients overall had LVH; among these, 56.3% had concentric LVH, 30.1% had eccentric LVH, 6.8% had concentric remodeling and only 6.8% of the patients had normal LV geometry. However, in our study, the proportion of centripetal hypertrophy (28.0%) was slightly higher than that of eccentric hypertrophy (25.1%). The cause of this outcome is obscure, but most probably satisfactory volume and BP control played a central role. In addition, the incidence of cognitive impairment in centripetal hypertrophy was higher than that in eccentric hypertrophy, but the incidence was not statistically significant.

One possible mechanism for the relationship between LVH and cognitive dysfunction is the presence of white matter damage [33]. In individuals with cardiovascular risk factors, white matter damage has been found in regions of the dorsolateral prefrontal and anterolateral orbital circuits [58]. Dysfunction of these frontal subcortical circuits is characterized by impaired executive function and memory [59]. These results are in agreement with our study. The LVH group of patients undergoing PD had significant differences in visuospatial/executive function, language, and delayed recall (all *P* < 0.001).

Zhong et al.^[43]^demonstrated a significant association between middle-aged CAS and cognitive function. Unfortunately, we found that the incidence of atherosclerosis in patients undergoing PD was significantly different between the CI and NCI groups, but there was no significant association between atherosclerosis and CI after adjusting for confounding factors. This consideration may be related to the insufficient sample size and cognitive scoring tests, and further large-scale, multicenter studies are needed to draw definite conclusions.

Similar to the general population, age, education level, and diabetes mellitus are related to the cognitive degree of patients undergoing PD. However, we also found that hsCRP, albumin, and hemoglobin were independently associated with CI in patients undergoing PD. This is similar to the study of Stivelman et al. [60]. Higher albumin and hemoglobin levels are protective factors for cognitive function [61, 62]. The underlying mechanism of albumin may lie in its role as an antioxidant during inflammation, reducing oxidative damage in the pathogenesis of CI.In addition, the hemoglobin of patients with peritoneal dialysis in this study (86.00 ± 13.62 g/L) was slightly lower than that in Yin [63] et al.’s study (93.0 ± 19.6), which was considered to be related to the poor compliance of patients with peritoneal dialysis in this study, low return rate, and insufficient dosage of erythropitin.

The strength of this study was that it was the first to reveal an association between LVH and CAS and CI in patients undergoing PD. There are some limitations to the study, however. First, as a single-center study, the small sample size may limit its generality. Second, this study has a cross-sectional design, so causal inferences cannot be made. In addition, we only applied MoCA to assess cognition. MoCA is a short screening tool that does not fully assess cognitive function the way neuropsychological tests do. The use of magnetic resonance imaging is recommended to identify asymptomatic cerebral infarction, microbleeds, and white matter disease.

## Conclusions

We showed that LVH is independently associated with CI in patients undergoing PD, especially in visuospatial/executive functions, delayed memory, language, and attention. Although the mechanism underlying the association between LVH and CI in patients undergoing PD has not been clarified, new directions for further research are proposed. Early identification of LVH has significant benefits in reducing the prevalence of CI. In addition, CAS was not significantly associated with CI after adjusting for confounding factors.

## Supplementary Information

**Additional file 1: Table S1.** Differences in clinical characteristics between PD Patients with and without CAS

**Additional file 2: Table S2.** Differences in clinical characteristics between PD Patients With and without CI

**Additional file 3: Table S3.** Multivariate logistic regression analysis of factors associated with cognitive impairment in peritoneal dialysis

## Data Availability

All data generated or analysed during this study are included in this article and its supplementary information files. Further enquiries can be directed to the corresponding author.

## Abbreviations

ALT: Alanine transaminase
AST: Aspartate aminotransferase
BMI: Body mass index
BP: Blood pressure
CAS: Carotid atherosclerosis
Ccr: Creatinine clearance
CVD: Cardiovascular disease
CI: Cognitive impairment
EF: Ejection fraction
ESR: Erythrocyte sedimentation rate
hsCRP: High-sensitivity C-reactive protein
LVH: Left ventricular hypertrophy
LVMI: Left ventricular mass index
MOCA: Montreal Cognitive Assessment
PD: Peritoneal dialysis
PTH: Parathyroid hormone
RWT: Relative wall thickness
TSH: Thyroid stimulating hormone
