# Supplementary material for: Left ventricular hypertrophy, carotid atherosclerosis, and cognitive impairment in peritoneal dialysis patients
Source: BMC Cardiovasc Disord. 2023 Mar 9;23:127. doi: 10.1186/s12872-023-03130-0 (PMC9996916; doi:10.1186/s12872-023-03130-0)
Supplement: Supplementary file 1 — Additional file 1: Table S1. Differences in clinical characteristics between PD Patients with and without CAS [file 12872_2023_3130_MOESM1_ESM.docx]

Supplementary Table 1:Differences in Clinical Characteristics Between PD Patients With and Without CAS.

| Characteristics | NCAS（n=96） | CAS（n=111） | T/Z/x2 | P |
| --- | --- | --- | --- | --- |
| Age,(years ) | 51.8±15.91 | 52.42±14.08 | -0.298 | 0.766 |
| BMI,(kg/m2) | 23.17±3.72 | 22.88±3.58 | 0.571 | 0.569 |
| Males,n(%) | 44（45.8） | 62（55.9） | 2.070 | 0.150 |
| Diuretics,n(%) | 10（10.4） | 14（12.6） | 0.242 | 0.623 |
| Beta-blockers, n(%) | 38（39.6） | 60（54.1） | 4.324 | 0.038 |
| Calcium channel blockers,n(%) | 82（85.4） | 97（87.4） | 0.171 | 0.679 |
| ACE/ARB inhibitors,n(%) | 17（17.7） | 17（15.3） | 0.215 | 0.643 |
| Cardiovascular disease, n(%) | 13（13.5） | 12（10.8） | 0.362 | 0.548 |
| Hypertension,n(%) | 92（95.8） | 107（96.4） | 0.044 | 0.834 |
| Diabetes mellitus,n (%) | 22（22.9） | 37（33.3） | 2.741 | 0.098 |
| Smoking History (%) | 35（36.5） | 58（52.3） | 5.190 | 0.023 |
| Systolic BP (mmHg) | 144.35±20.03 | 143.99±22.59 | 0.122 | 0.903 |
| Diastolic BP (mmHg) | 86.93±12.54 | 86.67±14.36 | 0.138 | 0.890 |
| Pulse pressure (mmHg) | 57.43±15.68 | 57.32±15.68 | 0.047 | 0.963 |
| [Total](javascript:;) [cholesterol](javascript:;) ,(mmol/L) | 4.35±0.97 | 4.4±1.03 | -0.291 | 0.772 |
| Serum glucose,(mmol/L) | 4.82（4.37-5.57） | 4.89（4.35-5.43） | -0.066 | 0.947 |
| [triglyceride](javascript:;) ,(mmol/L) | 1.31（0.86-1.81） | 1.36（0.95-1.9） | -0.556 | 0.578 |
| HDL cholesterol (mmol/L) | 0.97（0.86-1.23） | 1（0.8-1.24） | -0.226 | 0.821 |
| LDL cholesterol (mmol/L) | 2.11±0.67 | 1.97±0.65 | 1.532 | 0.127 |
| LV ejection fraction (%) | 58.5（55-62） | 58（55-62） | -0.31 | 0.757 |
| MOCA score | 26（21-27） | 24（22-26） | -1.434 | 0.152 |
| MOCA<26 (%) | 45（46.9） | 71（64） | 6.102 | 0.014 |
| LVH,n(%) | 46（47.9） | 64（57.7） | 1.962 | 0.161 |
| LVMI,(g/m^2.7^) | 48.94（42.3-61.16） | 52.94（41.91-71.31） | -1.659 | 0.097 |

Note: Values for categorical variables are given as number (percentage); values for continuous variables, as mean ± standard deviation or median [interquartile range].

Abbreviations: BMI, body mass index;LVMI,Left Ventricular Mass Index.
