# Supplementary material for: Left ventricular hypertrophy, carotid atherosclerosis, and cognitive impairment in peritoneal dialysis patients
Source: BMC Cardiovasc Disord. 2023 Mar 9;23:127. doi: 10.1186/s12872-023-03130-0 (PMC9996916; doi:10.1186/s12872-023-03130-0)
Supplement: Supplementary file 2 — Additional file 2: Table S2. Differences in clinical characteristics between PD Patients With and without CI [file 12872_2023_3130_MOESM2_ESM.docx]

Supplementary Table 2:Differences in Clinical Characteristics Between PD Patients With and Without CI.

| Characteristics | NCI（n=91） | CI（n=116） | T/Z/x2 | P |
| --- | --- | --- | --- | --- |
| Age,(years ) | 44.57±13.30 | 58.07±13.41 | -7.214 | ＜0.001 |
| BMI,(kg/m2) | 23.03±4.01 | 23.00±3.34 | 0.055 | 0.956 |
| Males,n(%) | 48（52.7） | 58（50） | 0.154 | 0.695 |
| Education (%) |  |  | 48.242 | ＜0.001 |
| ≤Elementary school | 11（12.1） | 61（52.6） | - | - |
| Middle school | 26（28.6） | 34（29.3） | - | - |
| High school | 27（29.7） | 12（10.3） | - | - |
| ＞High school | 27（29.7） | 9（7.8） | - | - |
| Primary cause of ESRD (%) |  |  | 1.305 | 0.521 |
| Glomerulonephritis | 56（61.5） | 72（62.1） | - | - |
| Diabetes | 10（11） | 18（15.5） | - | - |
| Other | 25（27.5） | 26（22.4） | - | - |
| Diuretics,n(%) | 11（12.1） | 13（1012） | 0.039 | 0.844 |
| Beta-blockers, n(%) | 46（50.5） | 52（44.8） | 0.670 | 0.413 |
| Calcium channel blockers,n(%) | 79（86.8） | 100（86.2） | 0.016 | 0.899 |
| ACE/ARB inhibitors,n(%) | 13（14.3） | 21（18.1） | 0.541 | 0.462 |
| Cardiovascular disease, n(%) | 7（7.7） | 18（15.5） | 2.940 | 0.086 |
| Hypertension,n(%) | 88（96.7） | 111（95.7） | 0.141 | 0.707 |
| Diabetes mellitus,n (%) | 16（17.6） | 43（37.1） | 9.502 | 0.002 |
| Smoking History (%) | 39（42.9） | 54（46.6） | 0.281 | 0.596 |
| Systolic BP (mmHg) | 142.53±21.61 | 145.44±21.22 | -0.972 | 0.332 |
| Diastolic BP (mmHg) | 87.54±15.02 | 86.20±12.23 | 0.69 | 0.491 |
| Pulse pressure (mmHg) | 54.99±14.07 | 59.24±16.59 | -1.995 | 0.052 |
| Duration of dialysis (months) | 7（5-26） | 8（5-16.8） | -0.367 | 0.713 |
| Hemoglobin,(g/L) | 91.26±15.05 | 81.79±10.74 | 5.072 | ＜0.001 |
| Glycated hemoglobin，(%) | 5.3（5-5.8） | 5.5（5.1-6） | -1.914 | 0.056 |
| TSH(μIU/mL) | 2.28（1.4-3.25） | 2.635（1.51-4.47） | -1.598 | 0.11 |
| Albumin,(g/L) | 36.07±4.51 | 31.66±4.51 | 6.200 | ＜0.001 |
| ALT,(/L) | 10.5（7.2-16.5） | 11（7.7-16） | -0.612 | 0.541 |
| AST,(u/L) | 13.9（11-18） | 15（12.03-18.93） | -1.573 | 0.116 |
| Serum glucose,(mmol/L) | 4.8（4.34-5.47） | 4.9（4.36-5.44） | -0.417 | 0.676 |
| serum urea nitrogen(mmol/L) | 19.20±9.34 | 20.42±7.55 | -1.04 | 0.300 |
| Serum creatinine,(umol/L) | 800.67±362.40 | 815.13±310.11 | -0.303 | 0.762 |
| [Uric Acid](javascript:;),(umol/L) | 399.46±114.82 | 419.53±92.59 | -1.357 | 0.177 |
| [Total](javascript:;) [cholesterol](javascript:;) ,(mmol/L) | 4.27±1.06 | 4.46±0.93 | -1.363 | 0.174 |
| [triglyceride](javascript:;) ,(mmol/L) | 1.36（0.84-1.94） | 1.29（0.91-1.77） | -0.566 | 0.572 |
| HDL cholesterol (mmol/L) | 0.96（0.85-1.22） | 1（0.83-1.263） | -0.343 | 0.732 |
| LDL cholesterol (mmol/L) | 2.05±0.68 | 2.01±0.65 | 0.436 | 0.663 |
| PTH (ng/L) | 236.78±146 | 278.1±215.89 | -1.566 | 0.119 |
| Calcium, (mmol/L) | 2.15±0.22 | 2.12±0.25 | 0.696 | 0.487 |
| Phosphorus, (mmol/L) | 1.88±0.56 | 1.83±0.97 | 0.499 | 0.618 |
| Potassium, (mmol/L) | 4.09±0.66 | 4.02±0.69 | 0.693 | 0.489 |
| Sodium, (mmol/L) | 140.4±2.8 | 139.93±3.09 | 1.141 | 0.255 |
| ESR(mm/h) | 25（15-42） | 29（16-45.8） | -1.007 | 0.314 |
| Hs-CRP(mg/L) | 3.54±2.12 | 5.58±3.09 | -5.635 | ＜0.001 |
| LV ejection fraction (%) | 59（55-63） | 58（55-61） | -1.183 | 0.237 |
| Total Kt/V | 1.98±0.33 | 1.9±0.41 | 1.451 | 0.148 |
| Ccr（ml/min） | 8.64（6.51-11.51） | 7.07（5.47-9.28） | -3.301 | 0.001 |
| LVH,n(%) | 28（30.8） | 82（70.7） | 32.636 | ＜0.001 |
| CAS,n(%) | 40（44） | 71（61.2） | 6.102 | 0.014 |
| LVMI,(g/m^2.7^) | 46.1（40.77-54.35） | 58.4（46.62-76.12） | -5.359 | ＜0.001 |

Note: Values for categorical variables are given as number (percentage); values for continuous variables, as mean ± standard deviation or median [interquartile range].

Abbreviations: BMI, body mass index; TSH,Thyroid Stimulating Hormone;.ALT, Alanine transaminase;AST,Aspartate aminotransferase;PTH,Parathyroid Hormone;ESR,[erythrocyte sedimentation rate](javascript:;);hsCRP,high-sensitivity C-reactive protein;Ccr,[creatinine clearance rate](javascript:;);LVH,left ventricular hypertrophy;CAS,carotid atherosclerosis;LVMI,Left Ventricular Mass Index.
