# Supplementary material for: Left ventricular hypertrophy, carotid atherosclerosis, and cognitive impairment in peritoneal dialysis patients
Source: BMC Cardiovasc Disord. 2023 Mar 9;23:127. doi: 10.1186/s12872-023-03130-0 (PMC9996916; doi:10.1186/s12872-023-03130-0)
Supplement: Supplementary file 3 — Additional file 3: Table S3. Multivariate logistic regression analysis of factors associated with cognitive impairment in peritoneal dialysis [file 12872_2023_3130_MOESM3_ESM.docx]

Supplementary Table 3:Multivariate logistic regression analysis of factors associated with cognitive impairment in peritoneal dialysis.

| Variable | OR | 95% CI | P value |
| --- | --- | --- | --- |
| Age,(years ) | 1.073 | 1.025-1.124 | 0.003 |
| Diabetes mellitus n (%) | 5.124 | 1.592-16.492 | 0.006 |
| Level of education |  |  | ＜0.001 |
| ≤Elementary school |  | Reference |  |
| Middle school | 0.182 | 0.042-0.792 | 0.023 |
| High school | 0.036 | 0.007-0.198 | ＜0.001 |
| ＞High school | 0.034 | 0.006-0.195 | ＜0.001 |
| Hemoglobin,(g/L) | 0.949 | 0.910-0.991 | 0.017 |
| Albumin,(g/L) | 0.814 | 0.723-0.917 | 0.001 |
| hsCRP,(mg/L) | 2.137 | 1.557-2.935 | ＜0.001 |
| LVH n(%) | 10.087 | 2.966-34.307 | ＜0.001 |

Abbreviations: hsCRP, high-sensitivity C-reactive protein ;LVH, [left ventricular hypertrophy](javascript:;).
